# Usage and Usability of a National e-Library for Chemotherapy Regimens: Mixed Methods Study

**DOI:** 10.2196/33651

**Published:** 2022-02-17

**Authors:** AnnSofie Fyhr, Johanna Persson, Åsa Ek

**Affiliations:** 1 Regional Cancer Centre South Region Skåne Lund Sweden; 2 Ergonomics and Aerosol Technology, Department of Design Sciences Faculty of Engineering Lund University Lund Sweden

**Keywords:** chemotherapy regimens, user evaluation, standardization, patient safety, chemotherapy, safety, usability, e-library, medication errors

## Abstract

**Background:**

Accurate information about chemotherapy drugs and regimens is needed to reduce chemotherapy errors. A national e-library, as a common knowledge source with standardized chemotherapy nomenclature and content, was developed. Since the information in the library is both complex and extensive, it is central that the users can use the resource as intended.

**Objective:**

The aim of this study was to evaluate the usage and usability of an extensive e-library for chemotherapy regimens developed to reduce medication errors, support the health care staff in their work, and increase patient safety.

**Methods:**

To obtain a comprehensive evaluation, a mixed methods study was performed for a broad view of the usage, including a compilation of subjective views of the users (web survey, spontaneous user feedback, and qualitative interviews), analysis of statistics from the website, and an expert evaluation of the usability of the webpage.

**Results:**

Statistics from the website show an average of just over 2500 visits and 870 unique visitors per month. Most visits took place Mondays to Fridays, but there were 5-10 visits per day on weekends. The web survey, with 292 answers, shows that the visitors were mainly physicians and nurses. Almost 80% (224/292) of respondents searched for regimens and 90% (264/292) found what they were looking for and were satisfied with their visit. The expert evaluation shows that the e-library follows many existing design principles, thus providing some useful improvement suggestions. A total of 86 emails were received in 2020 with user feedback, most of which were from nurses. The main part (78%, 67/86) contained a question, and the rest had discovered errors mainly in some regimen. The interviews reveal that most hospitals use a computerized physician order entry system, and they use the e-library in various ways, import XML files, transfer information, or use it as a reference. One hospital without a system uses the administration schedules from the library.

**Conclusions:**

The user evaluation indicates that the e-library is used in the intended manner and that the users can interact without problems. Users have different needs depending on their profession and their workplace, and these can be supported. The combination of methods applied ensures that the design and content comply with the users’ needs and serves as feedback for continuous design and learning. With a broad national usage, the e-library can become a source for organizational and national learning and a source for continuous improvement of cancer care in Sweden.

## Introduction

Chemotherapy treatments are highly complex, and errors may cause serious harm among patients with cancer, which is a particularly sensitive group owing to impaired tolerance [1,2]. Errors in the chemotherapy process occur at all stages of medication use [2]. However, the prescribing stage plays a key role in the creation of chemotherapy errors [3,4]. Therefore, the use of computerized physician order entry (CPOE) or chemotherapy prescription clinical decision support systems (CDSSs) is recommended [5,6]. Multiple stakeholders, physicians, nurses, and pharmacists, but also patients and their relatives, are involved in the process. This adds to the risk picture, which emphasizes the importance of accurate and timely information about dosages, sequence of therapies, supportive medications, and duration of treatment [7]. Therefore, a common source providing this information was needed. This paper presents a newly developed e-library for chemotherapy regimens with a focus on results from a usage and usability study.

In Sweden, descriptions of chemotherapy regimens were earlier developed and compiled locally within health care organizations. This was mostly because Sweden, with its 10 million inhabitants, is divided into 21 county councils, representing different geographical regions, all having far-reaching autonomy regarding the planning, financing, and operation of the region’s health care. In total, there are 17 oncology clinics in Sweden, which manage chemotherapy regimens, of which 7 are located at university hospitals. Additionally, there are several smaller units at other health care clinics, which also manage regimens. The many autonomous clinics have resulted in the same chemotherapy treatments occurring under different names and with different dosages causing uncertainty and risks for mix-ups. To overcome these uncertainties and risks, a national knowledge source for regimens (an e-library) was developed, with standardized nomenclature and content in chemotherapy regimens, also facilitating the exchange of information between hospitals, CPOE systems, and patients.

A standardized national source for chemotherapy regimens can constitute a preventive safety barrier function in the chemotherapy process [8]. Standardization of workflow processes, prescribing, preparation, dispensing, and administration are recommended as safeguards against medication errors [9,10]. Standardized order sets [11], standardized design and architecture [12], and standardized protocols and dosing [13] are beneficial when implementing CPOE systems. In 2020, the HemOnc group (working with a collaborative web-based knowledge platform for oncology professionals in the United States) published a proposal for a standardized nomenclature for chemotherapy regimens that were also compared to the thesaurus of the US National Cancer Institute [14]. In a recently published article, a review of attempts to standardize chemotherapy nomenclature is presented together with recommendations from a European expert panel of oncology pharmacists [15]. In this case, standardization is one way to ensure that all involved health care units have access to a standardized nomenclature and the latest evidence on chemotherapy treatments, which also allows for increased patient safety.

For the national e-library to serve as the intended safety barrier function, it needs to be designed and developed in accordance with user and usability criteria. Shulman et al [16] discusses principles for oncology eHealth records and claims that such systems should be designed to perform logically and straightforwardly, be user-friendly, and always be available to users. The same is applicable for a web-based resource. Based on such an approach the e-library was developed in a user-centered process and has been available since 2015 [17]. Representatives from different user groups were involved throughout the development process, including oncology nurses, physicians, hospital pharmacists, and patients with cancer.

**Figure 1:**
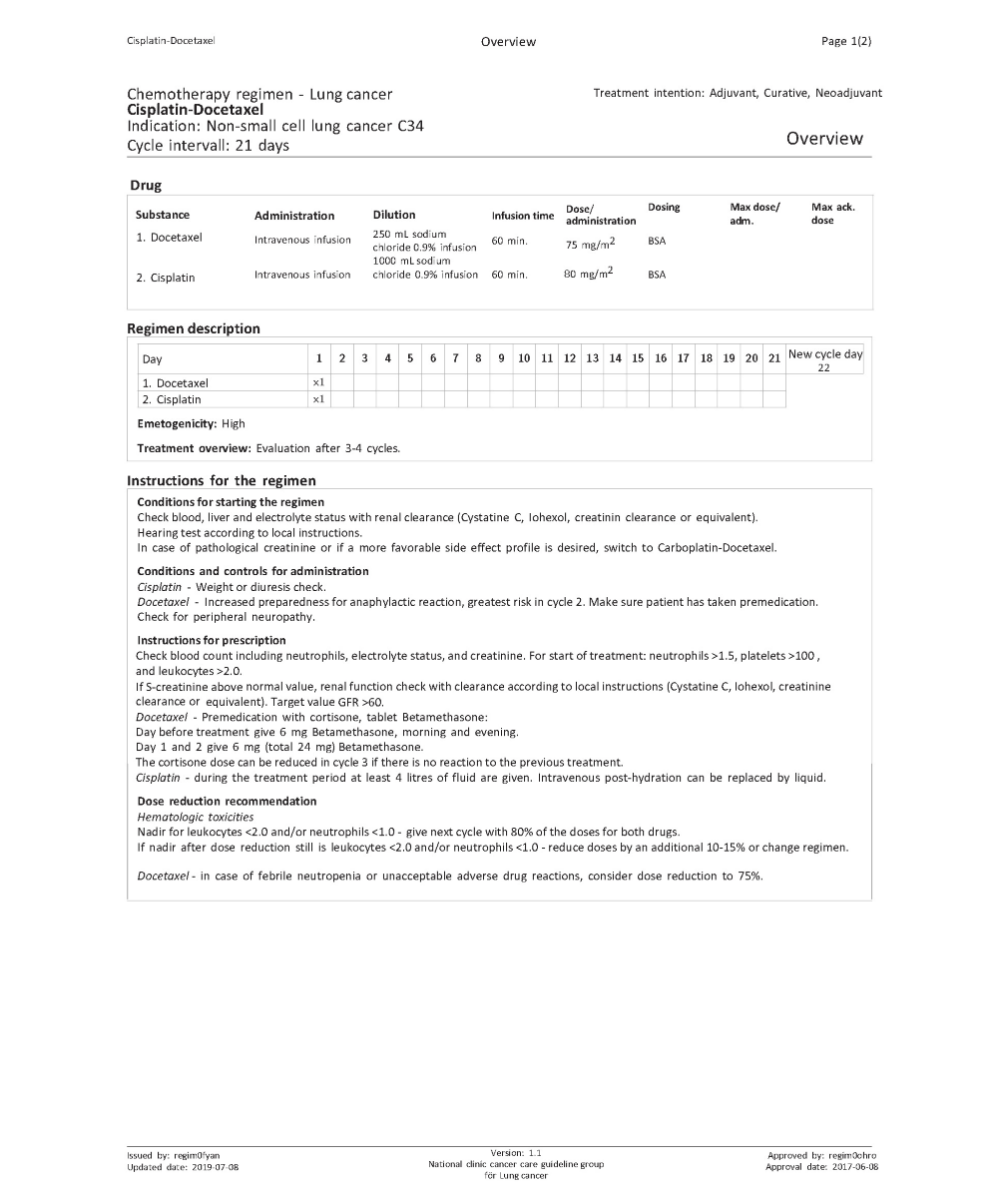
Overview of the Cisplatin-Docetaxel regimen for lung cancer. BSA: body surface area, GFR: glomerular filtration rate.

The national e-library [18] contains the following parts: (1) basic facts containing important medical and pharmaceutical information on drugs; (2) regimens presented per diagnosis with an overview (including instructions, precautions, and recommendation for dose reduction), adverse drug reactions (ADRs), and a detailed administration schedule; (3) information sheets for patients per regimen, providing a short description of the treatment and the most common or important ADRs with advice for self-treatment and when to contact the hospital; (4) support documents for health care professionals; and (5) newsletters published after updates of the e-library. Part of a regimen is shown in Figure 1. The primary users are physicians, nurses, and pharmacists. They can access the information in the e-library for reading, printing, or downloading XML files for the CPOE systems used in Sweden. The patients gain access to the information sheets per regimen through their nurse.

Since the information in the library is both complex and extensive, it is central that the users can utilize the resource as intended. Otherwise, it might not be the source for improved performance and quality in cancer care that is envisioned. The aim of this study was to evaluate the usage and usability of the e-library for chemotherapy regimens developed to reduce medication errors and increase patient safety. This paper presents the results from the evaluation, which will also serve as input for continuous development and improvement.

## Methods

**Figure 2:**
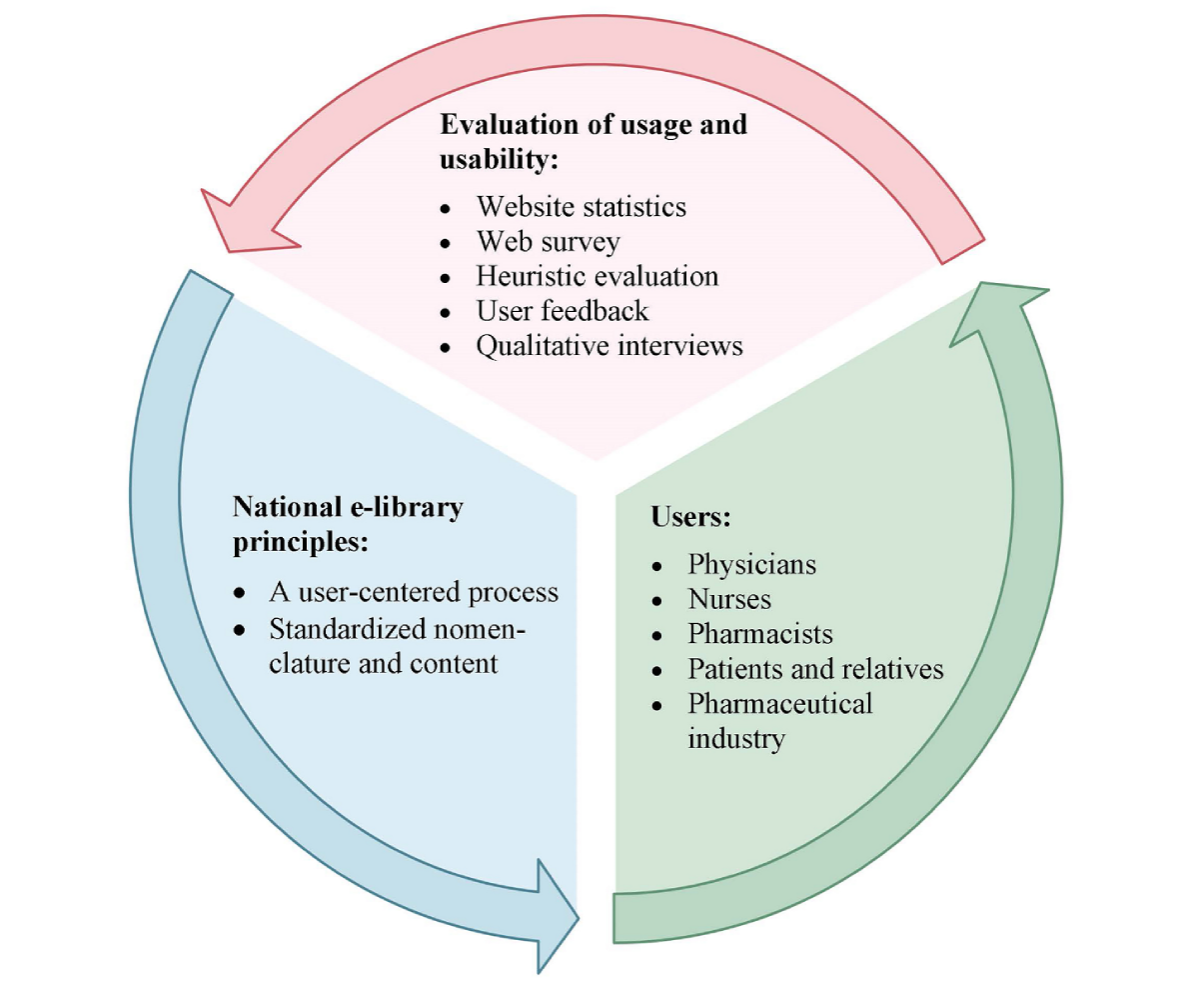
Evaluation of the e-library using multiple methods to conclude its intended usage and usability and to iteratively improve the resource.

A combination of methods was chosen to obtain a comprehensive view of the usage, including a compilation of subjective views of the users, analysis of statistics from the website, and an expert evaluation of the usability of the e-library [19]. The results will help understand where further development is needed or will have the most impact (Figure 2). The evaluation consisted of five parts:

1. Website statistics collected from May to December 2020 show the number of users in total, unique users, when they visit the e-library, and from where the visitors were.
2. A web survey for people visiting the e-library was conducted during January 2020. The survey consisted of questions about the visitor’s role and function, what information they were looking for, how pleased they were with the visit, and if they had suggestions of improvements.
3. A heuristic evaluation of the user interface [20] was performed in June 2020 to identify details in the user interface design that could be improved to increase usability.
4. Spontaneous user feedback was collected in the form of emails sent to the development team or project leader during 2020, which was obtained from users presenting their role and question or suggestions.
5. Qualitative interviews with 4 nurses, 3 physicians, and 3 pharmacists from various regions in Sweden were performed at the end of 2020 and at the beginning of 2021. As the intention is that the patients should get regimen-related information through their nurse and not through the e-library, no patient interviews were conducted. Interview questions concerned what parts of the e-library they used, how they used it, their experiences of using the e-library in their work compared to how they worked before, and their expectations for further development. The interviews were transcribed verbatim, and a process of transcript analysis was applied to identify themes.

## Results

### Website Statistics

During the last 8 months of 2020, the average number of visits to the website per month was just above 2500, and the number of unique visitors was 870. Most visitors were from Sweden, with some users from other countries in northern Europe, especially Scandinavia. Visitors from Sweden’s 3 largest cities (Stockholm, Gothenburg, and Malmoe) accounted for more than 50% of all visits. Most visits took place on Mondays to Fridays, but there were 5-10 visits per day on weekends.

### Web Survey

From the survey with 292 answers, it became clear that the visitors were mainly physicians (49%, 144/292) and nurses (36%, 104/292), and, to some extent, pharmacists (7%, 21/292). The rest were from the pharmaceutical industry and others. Almost 80% (224/292) stated that they searched for regimens and 90% (264/292) stated that they found what they were looking for. Most of the users were satisfied with their visit. The less satisfied visitors (8%, 22/292) lacked the regimens or diagnostic areas that they were looking for. There were 32 comments, 18 of which asked for more regimens and information sheets for patients. The rest of the comments concerned the design of the survey or were positive reviews.

### Heuristic Evaluation

The heuristic evaluation was performed in accordance with the Nielsen and Molich’s [20] framework, which implies that a product or system is evaluated in a structured way following ten predefined usability heuristics: (1) visibility of system status; (2) match between systems and the real world; (3) user control and freedom; (4) consistency and standards; (5) error prevention; (6) recognition rather than recall; (7) flexibility and efficiency of use; (8) aesthetic and minimalist design; (9) recognize, diagnose, and recover from errors; and (10) help and documentation. The results show that the e-library follows many of the existing design principles; therefore. it could be concluded to be usable in its current form. Nevertheless, some improvement suggestions were identified, such as moving patient information sheets to a separate tab in the menu, improving the search function by providing suggestions, and improving the start page by moving newsletters to a separate tab.

### Spontaneous User Feedback

A total of 86 emails with spontaneous user feedback were received from nurses (50%, 43/86), pharmacists (20%, 17/86), physicians (17%, 15/86), the pharmaceutical industry (8%, 7/86), and others (5%, 4/86). Of the emails, 78% (67/86) concerned questions such as “When will regimens arrive in a certain diagnostic group?” “How long should the drug temozolomide be given before starting radiation therapy?” In 19 of 86 (22%) emails, the sender had discovered errors in the e-library, which mainly concern the regimens, such as patient information sheets where there is a lack of a certain side effect or that the incorrect “treatment intention” had been chosen. All emails were answered, and in cases where there was incorrect or unclear information in the e-library’s documents, it was rectified.

### Qualitative Interviews

Interview results showed that most hospitals used a CPOE system for their prescription and administration (3 systems exist). One hospital has no system; instead, they use the administration schedule from the e-library. Those with a CPOE system use the library in various ways, including importing the XML files, reading the information in the library and then transferring the information to their CPOE system, or using the library as a reference to check their information. The newsletter is published regularly on the e-library’s website, with information on new regimens or basic facts, and detailed information about changes in the regimens was appreciated by most of the interviewees, but not all of them had noticed them. The basic facts are used in various ways depending on profession; the pharmacists focus on preparation, shelf-life, and references to stability studies, the nurses focus on ADRs, and the physicians focus on new drugs. The nurses, especially, were interested in the patient information sheets that are available for most of the regimens. Most hospitals already had their patient information sheets but will switch to the ones in the e-library. The available supporting documents for health care professionals, and especially “Management of side effects associated with immunotherapy with checkpoint inhibitors,” are indicated by the nurses and physicians as very useful. Among the expectations for further development was a harmonization of regimens used in different diagnostic areas; for example, the same amount of infusion fluid and infusion times for the same drug.

## Discussion

### Principal Findings

The results from the user evaluation indicate that the e-library can be concluded to be used in the intended manner, and the users do not have any problems interacting with the knowledge source. From the web surveys and interviews, it becomes clear that it is the content that the users focus on. The usability of the website is not addressed by the users, which may be interpreted as subjective satisfaction in the interaction with the system. The heuristic evaluation showed that there were minor usability issues that should be addressed to improve the overall usability of the website. The web survey showed that the users substantially are satisfied with their visit. Most of the users found what they were looking for, and their main feedback was a desire for more regimens, more diagnostic areas to be covered, and more patient information sheets, which are continuously added and updated. The expressed needs from the users are useful to understand which areas and regimens should be prioritized in the development work. The number of visits indicates that the resource is used extensively, and the geographical span shows that the e-library has emerged as the national resource it is intended to be. Users from almost all Swedish regions exist, although the 3 largest cities stand for the main usage. However, the introduction of the library has proceeded fairly quickly, and it is believed that it is only a matter of time before all regions have adopted the use of the e-library.

The spontaneous user feedback shows clearly that the e-library is used. Contact with the users is vital, and websites generally facilitate a rapid and comprehensive means of knowledge dissemination [7]. Emails from users reporting incorrectness are gratefully received, and they show that errors can slip through despite several checks. The interviews revealed various ways to use the information in the regimens. Some use the XML files to import the regimen to their CPOE system, which is a way to ensure that the transmitted information is accurate. Those transferring the information explained that they had to adjust information in their CPOE system; therefore, is it easier to copy one of their regimens and implement the necessary changes to match the new regimen. Some clinics do not have any CPOE system yet, still relying on handwritten orders and documentation. To transfer information manually is always a risk for introducing errors. Users have different needs depending on whether they are doctors, nurses, or pharmacists and depending on whether they work at a university hospital or a smaller hospital. The e-library can support these different needs from the users.

Automated downloading of the regimens from the e-library to the CPOE systems could improve the process, ensuring updated information without manual handling. However, the CPOE systems do not support this yet, and the process and approval of the updated information at each local clinic must be adapted to the change.

### Limitations

This study uses a mix of methods to address the usage and usability of a web-based resource for chemotherapy regimens. One can always discuss which mix of methods is optimal, and the combination used here could certainly be extended to include additional ones. Cognitive walkthrough is one such method, which could have been adopted—it is an explicit and detailed procedure to simulate a uses’ problem-solving process at each step through the dialogue with the system having potentially provided additional input in evaluating its usability [21].

The qualitative interviews revealed that nurses have started to replace their old patient information sheets with the ones in the e-library. In a follow-up study, it would be relevant to include patients in the evaluation process.

The study is performed in a geographically delimited system (Sweden), but the article contributes with working methods for how user-driven development contributes to a more standardized working method, which could also provide safer care. That knowledge is useful beyond the Swedish healthcare context. Finally, a longer-term evaluation is required to gain good insight into how the system is used and to evaluate how it contributes to increased safety in cancer care.

### Conclusions

The comprehensive user evaluation conducted is an important part of continuing the user-centered process that started already during the development of the e-library. Multiple evaluation methods complement one another by providing input from several perspectives (ie, expert or user, subjective or objective) that may be triangulated and hence identify critical design aspects and user needs [19]. The combination of methods applied in the evaluation presented in this paper included both objective usage statistics, expert methods for usability, and the users’ subjective feedback through a web survey and qualitative interviews. This ensures that the design and content of the e-library comply with the users’ needs and works as feedback for continuous development and learning [22]. Thus, the evaluation is a vital part assuring that the e-library act as a safety barrier, is well designed and that general design flaw is avoided, a design flaw that otherwise could create new risks in the chemotherapy process. The evaluation will contribute to a deeper understanding of users’ judgements about the library content and help to develop strategies for increasing the national usage of the library. With a broad national usage, the e-library can become a source for organizational and national learning and a source for continuous improvement of cancer care in Sweden.

## Abbreviations

ADR: adverse drug reaction
CDSS: clinical decision support system
CPOE: computerized physician order entry

## References

[1] Müller T (2003). Typical medication errors in oncology: analysis and prevention strategies. Onkologie.

[2] Weingart SN, Zhang L, Sweeney M, Hassett M (2018). Chemotherapy medication errors. Lancet Oncol.

[3] Serrano-Fabiá A, Albert-Marí A, Almenar-Cubells D, Jiménez-Torres NV (2010). Multidisciplinary system for detecting medication errors in antineoplastic chemotherapy. J Oncol Pharm Pract.

[4] Fyhr A, Akselsson R (2012). Characteristics of medication errors with parenteral cytotoxic drugs. Eur J Cancer Care (Engl).

[5] Kullberg A, Larsen J, Sharp L (2013). 'Why is there another person's name on my infusion bag?' Patient safety in chemotherapy care - a review of the literature. Eur J Oncol Nurs.

[6] Rahimi R, Moghaddasi H, Rafsanjani KA, Bahoush G, Kazemi A (2019). Effects of chemotherapy prescription clinical decision-support systems on the chemotherapy process: A systematic review. Int J Med Inform.

[7] Warner JL, Cowan AJ, Hall AC, Yang PC (2015). HemOnc.org: A Collaborative Online Knowledge Platform for Oncology Professionals. J Oncol Pract.

[8] Hollnagel E (2004). Barriers and Accident Prevention.

[9] Goldspiel B, Hoffman JM, Griffith NL, Goodin S, DeChristoforo R, Montello CM, Chase JL, Bartel S, Patel JT (2015). ASHP guidelines on preventing medication errors with chemotherapy and biotherapy. Am J Health Syst Pharm.

[10] Huertas-Fernández MJ, Martínez-Bautista MJ, Rodríguez-Mateos ME, Zarzuela-Ramírez M, Muñoz-Lucero T, Baena-Cañada JM (2017). Implementation of safeguards to improve patient safety in chemotherapy. Clin Transl Oncol.

[11] Classen D, Bates DW, Denham CR (2010). Meaningful use of computerized prescriber order entry. J Patient Saf.

[12] Kukreti V, Cosby R, Cheung A, Lankshear S, ST Computerized Prescriber Order Entry Guideline Development Group (2014). Computerized prescriber order entry in the outpatient oncology setting: from evidence to meaningful use. Curr Oncol.

[13] Dabliz R, Poon SK, Ritchie A, Burke R, Penm J (2021). Usability evaluation of an integrated electronic medication management system implemented in an oncology setting using the unified theory of the acceptance and use of technology. BMC Med Inform Decis Mak.

[14] Rubinstein SM, Yang PC, Cowan AJ, Warner JL (2020). Standardizing Chemotherapy Regimen Nomenclature: A Proposal and Evaluation of the HemOnc and National Cancer Institute Thesaurus Regimen Content. JCO Clin Cancer Inform.

[15] Terkola R, Bardin C, Lizeaga Cundin G, Zeinab N, Crul M (2021). Identifying options for oncology therapy regimen codification to improve standardization-combined results of an expert panel and a review. J Clin Pharm Ther.

[16] Shulman LN, Miller RS, Ambinder EP, Yu PP, Cox JV (2008). Principles of Safe Practice Using an Oncology EHR System for Chemotherapy Ordering, Preparation, and Administration, Part 1 of 2. J Oncol Pract.

[17] Fyhr A, Borell J, Jerkeman M, Ek Å (2020). National e-library for standardized chemotherapy regimens. Acta Oncol.

[18] Regionala Cancercentrum i Samverkan.

[19] Price M, Weber J, Bellwood P, Diemert S, Habibi R, Lau F, Kuziemsky C (2017). Evaluation of eHealth System Usability and Safety. Handbook of eHealth Evaluation: An Evidence-based Approach.

[20] Nielsen J, Molich R (1990). Heuristic evaluation of user interfaces.

[21] Nielsen J, Mack RL (1994). Usability Inspection Methods.

[22] Carayon P, Salwei ME (2021). Moving toward a sociotechnical systems approach to continuous health information technology design: the path forward for improving electronic health record usability and reducing clinician burnout. J Am Med Inform Assoc.

